# Cardioplegia strategies in ALCAPA surgery: A narrative review on practical comparison of techniques

**DOI:** 10.1051/ject/2026013

**Published:** 2026-09-15

**Authors:** Youssef El Dsouki, Gopikrishnan Thalapathy, Ignazio Condello

**Affiliations:** 1 Faculty of Health, Medicine and Life Sciences, Cardiovascular Research Institute Maastricht (CARIM), Maastricht University P. Debyelaan, 25 6202 AZ Maastricht The Netherlands; 2 Sheikh Khalifa Medical City Abu Dhabi United Arab Emirates; 3 School of Medicine and Surgery, University of Varese Insubria, University of Insubria Via Ravasi, 2 21100 Varese VA Italy

**Keywords:** Cardioplegia, Myocardial protection, Congenital heart surgery, Antegrade perfusion, Pediatric cardiac surgery

## Abstract

Anomalous left coronary artery from the pulmonary artery (ALCAPA) is a rare yet life-threatening congenital cardiac anomaly in which the left coronary artery originates from the pulmonary trunk, leading to severe myocardial ischemia, infarction, and progressive heart failure if untreated. Definitive management requires surgical reimplantation of the anomalous artery into the aorta, supported by meticulously tailored cardioplegia to protect the vulnerable pediatric myocardium, whose perfusion is further complicated by right-to-left coronary collaterals. This manuscript reports the findings of a targeted narrative review of operative videos, technical tutorials, and institutional protocols, synthesising practical knowledge on myocardial protection strategies for ALCAPA repair. Three techniques emerged as the most widely adopted and technically distinct: (1) simultaneous aortic and pulmonary-root administration of cold Custodiol^®^, providing prolonged single-dose crystalloid protection; (2) antegrade cold-blood cardioplegia supplemented by direct left-coronary ostial perfusion after main pulmonary artery transection, allowing staged protection during extended cross-clamp times; and (3) a classical normothermic blood protocol with repeated root doses and routine half-dose ostial delivery, offering dynamic electrolyte control and familiarity to most congenital teams. For each strategy, we summarise precise indications, operative setup, perfusion pressures, dosing schedules, and practical tips for managing collateral-driven early electrical activity. By distilling evidence and expert practice into a concise technical comparison, this narrative review provides congenital cardiac surgeons with an up-to-date, step-by-step reference for safe, effective myocardial protection in ALCAPA repair.

## Introduction

**Figure 1:**
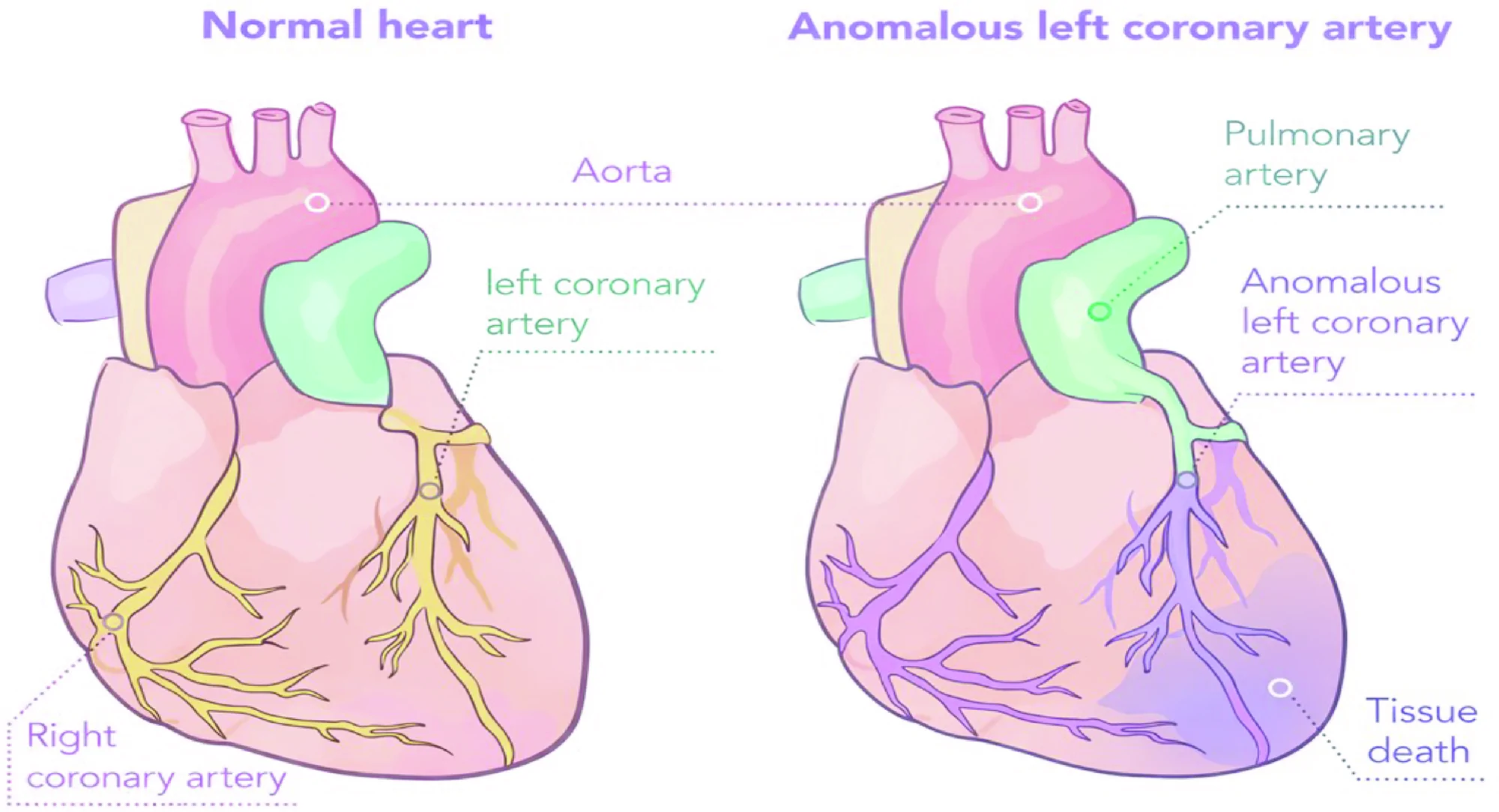
Comparison between normal coronary anatomy and anomalous origin of the left coronary artery from the pulmonary artery (ALCAPA). In ALCAPA, myocardial perfusion of the left ventricle depends on low-pressure pulmonary flow and right-to-left coronary collaterals, predisposing to ischemia and tissue injury.

Anomalous left coronary artery from the pulmonary artery (ALCAPA), also known as Bland-White-Garland syndrome, is a rare but potentially fatal congenital anomaly in which the left coronary artery originates abnormally from the main pulmonary artery rather than the aorta. It is estimated to occur in approximately 1 in 300,000 live births, accounting for 0.25–0.5% of all congenital heart defects [1]. The condition leads to a significant mismatch between myocardial oxygen supply and demand due to the low-pressure, desaturated pulmonary circulation providing insufficient perfusion to the left ventricular myocardium. During the neonatal period, as pulmonary vascular resistance decreases, the pressure gradient across the coronary circulation reverses, causing a coronary steal phenomenon. This further compromises myocardial oxygenation, often resulting in progressive ischemia, infarction, left ventricular dysfunction, mitral regurgitation, and heart failure. If left untreated, the mortality rate in infancy exceeds 90% [2]. In patients who survive into later infancy or childhood, compensatory collateral circulation from the right coronary artery may partially sustain myocardial viability. However, this collateral supply is hemodynamically inefficient and cannot fully prevent ischemic damage or arrhythmias, emphasizing the urgency of timely surgical correction. The cornerstone of surgical management is anatomical repair, most commonly through direct reimplantation of the anomalous LCA into the aortic root to re-establish a dual-coronary system. However, the distinct coronary anatomy in ALCAPA, marked by retrograde collateral flow, dilated right coronary artery, and potential ventricular scarring, presents unique challenges for myocardial protection during cardiopulmonary bypass. Standard cardioplegia strategies may be insufficient, as traditional antegrade delivery through the aortic root may not adequately reach the left coronary territory, especially prior to pulmonary artery transection [3, 4]. Therefore, myocardial protection in ALCAPA repair must be adapted to ensure homogeneous cardioplegia delivery to both coronary systems, prevent inadequate perfusion of the left ventricular myocardium, and manage early return of electrical activity due to right-to-left collateral circulation [5]. Tailored strategies involving simultaneous dual-root delivery, ostial perfusion, and controlled perfusion pressures are crucial in optimizing surgical outcomes and minimizing perioperative myocardial injury [6]. Several cardioplegia delivery strategies have been described for myocardial protection during ALCAPA repair, reflecting the wide anatomical variability of the condition and the absence of a universally accepted standard protocol. Reported approaches include single-dose crystalloid cardioplegia, cold or warm blood cardioplegia with antegrade, retrograde, or selective ostial delivery, as well as hybrid techniques combining multiple routes of administration. The choice of strategy is largely influenced by institutional experience, anticipated cross-clamp duration, availability of perfusion resources, and the need to address competitive right-to-left coronary collateral flow. For the purpose of this narrative review, three cardioplegia strategies were selected because they represent the most commonly adopted, technically distinct, and conceptually complementary approaches currently used in clinical practice. Specifically, these techniques encompass: (1) a single-dose crystalloid strategy based on simultaneous dual-root delivery; (2) a staged cold blood approach incorporating selective left coronary ostial perfusion; and (3) a classical normothermic blood protocol emphasizing metabolic flexibility and repeated dosing. Together, these strategies capture the full spectrum of myocardial protection philosophies applied to ALCAPA repair and allow a practical comparison relevant to most congenital cardiac surgery programs (Figure 1).

## Materials and methods

This technical report is the result of a structured narrative review focused on myocardial protection strategies for the surgical repair of ALCAPA. The primary aim was to identify, describe, and compare cardioplegia techniques that address the anatomical complexity and physiological challenges posed by this rare congenital coronary anomaly.

### PICOT framework

#### Population

Patients with ALCAPA undergoing surgical repair (pediatric, neonatal, and adult congenital reports eligible).

#### Intervention

Cardioplegia delivery strategies tailored to ALCAPA anatomy (dual-root delivery, ostial perfusion, blood-based protocols, crystalloid-based protocols).

#### Comparator

Alternative cardioplegia strategies (route, temperature, composition, dosing/redosing).

#### Outcomes

Technical feasibility, myocardial arrest maintenance, and practical intraoperative considerations (e.g., early electrical activity, LV distension); clinical outcomes were not systematically extracted due to heterogeneity and the narrative scope.

#### Time

Studies published 2000–2024.

### Search strategy (PubMed/MEDLINE)

The PubMed search combined free-text and MeSH terms using Boolean operators: (“ALCAPA” OR “anomalous left coronary artery from the pulmonary artery” OR “Bland-White-Garland”) AND (“cardioplegia” OR “myocardial protection” OR “cardiac arrest” OR “cardiopleg*” OR “del Nido” OR “Custodiol” OR “HTK” OR “crystalloid cardioplegia” OR “blood cardioplegia” OR “warm blood cardioplegia” OR “cold blood cardioplegia”) AND (“congenital” OR “pediatric” OR “infant” OR “neonate” OR “adult congenital”). Filters: English language; publication years 2000–2024.

### Study selection

The PubMed search retrieved 43 records**.** Titles and abstracts were screened for relevance to myocardial protection strategies in ALCAPA repair or closely related congenital coronary anomalies. Full texts of potentially eligible articles were then reviewed to assess whether they provided actionable technical information on cardioplegia delivery, including route of administration, dosing or redosing schedule, perfusion pressure, temperature, and/or selective ostial perfusion. Articles lacking sufficient technical detail, papers not directly applicable to ALCAPA myocardial protection, and reports focused primarily on postoperative outcomes without operative cardioplegia description were excluded. After full-text assessment**,** 15 articles met the inclusion criteria and were used for the technical comparative synthesis. Three cardioplegia strategies currently in clinical use were selected for comparison:

1) Simultaneous aortic and pulmonary root infusion of cold Custodiol^®^, designed to achieve uniform myocardial protection in a single dose, particularly when standard antegrade delivery may not adequately perfuse the anomalous left coronary system [2, 6].
2) Antegrade cold blood cardioplegia with direct ostial perfusion, a staged approach providing targeted LCA perfusion through the pulmonary root after main pulmonary artery (MPA) transection [3, 7].
3) Institutional normothermic blood cardioplegia protocol, involving repeated antegrade aortic root administration, supplemented by a half-dose delivered into the LCA ostium after MPA opening [8, 9].

Each technique was analyzed according to delivery route, solution composition and temperature, perfusion pressure, dosing strategy, timing, and specific considerations for left coronary ostial perfusion. Particular attention was given to intraoperative challenges such as early return of electrical activity due to RCA–LCA collaterals, left ventricular distension, and the need for redosing during prolonged ischemic intervals. No clinical or outcome data were collected or analyzed; the objective remains purely technical and illustrative for surgical reference.

## Results

A targeted PubMed search yielded 43 records. After title/abstract screening and full-text evaluation for technical applicability to ALCAPA myocardial protection, 15 articles met the inclusion criteria, namely, they provided sufficiently detailed information on cardioplegia delivery route, dosing/redosing strategy, perfusion pressure, temperature, and/or selective ostial perfusion that could be directly translated into operative practice. These 15 sources underpin the comparative synthesis presented below [1–15].

*The simultaneous aortic- and pulmonary-root Custodiol*^®^ technique emerges as an excellent single-shot strategy. Across the selected literature, it consistently produced rapid, homogeneous arrest of both coronary beds, a decisive advantage in the presence of an anomalous left coronary origin and competitive collateral flow. Electromechanical silence was routinely maintained for 90–120 min without redosing thanks to the solution’s prolonged buffering capacity. This technique is best suited to cases with moderate cross-clamp times. Its main limitations, as highlighted in several of the reviewed studies, are the need for careful pressure titration to avoid left ventricular distension and the limited metabolic flexibility inherent to crystalloid cardioplegia. In contrast to repeated blood cardioplegia dosing, crystalloid-based strategies do not allow the same degree of intraoperative adjustment of potassium concentration, temperature, hematocrit, or buffering capacity [8, 9].

*The antegrade cold-blood cardioplegia protocol* with direct left-coronary ostial supplementation was the most versatile solution for longer or staged reconstructions. Five papers describe reliable left-ventricular protection when an initial aortic-root dose is followed by a selective infusion into the LCA ostium after main-pulmonary-artery transection. Scheduled redosing every 25–30 min extended the protective window without significant ventricular distension. The trade-off is technical: accurate ostial cannulation and synchronized timing between the two delivery circuits are essential to avoid washout or under-perfusion [10, 11].

**Figure 2:**
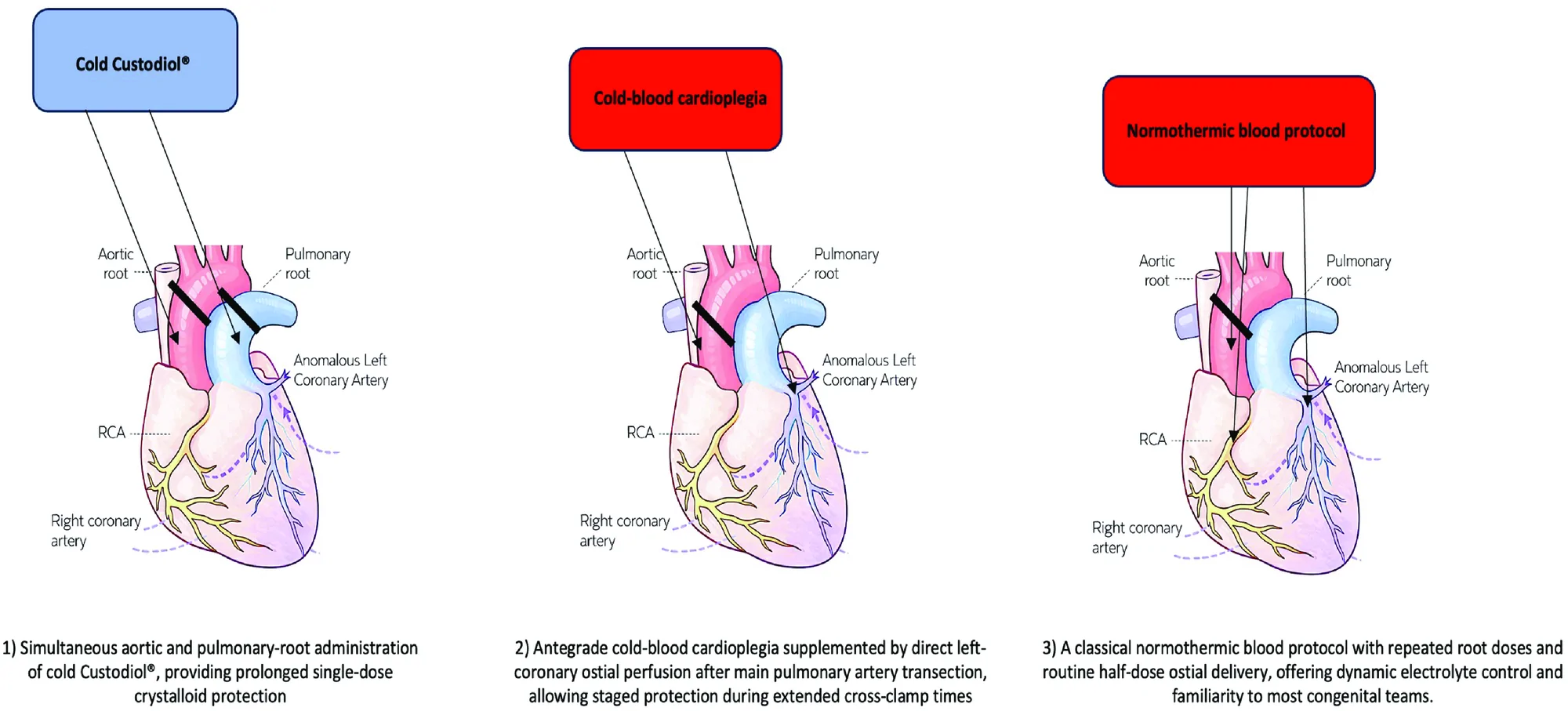
Schematic comparison of three cardioplegia strategies for ALCAPA repair: (1) dual-root cold Custodiol^®^ cardioplegia, (2) cold-blood cardioplegia with selective left coronary ostial perfusion, and (3) classical normothermic blood cardioplegia with repeated root dosing. The diagrams highlight cardioplegia delivery routes and the anomalous coronary anatomy characteristic of ALCAPA.

*The normothermic (warm) blood cardioplegia protocol* offered the greatest intra-operative adaptability, as documented in seven of the selected publications. Surgeons could titrate potassium concentration in real time and administer a terminal “hot-shot” reperfusion, which several authors associate with reduced reperfusion injury. The downside is workload: warm solutions demand boluses every 15–20 min and meticulous rhythm surveillance because vigorous right-to-left collaterals may trigger early electrical recovery, occasionally necessitating unscheduled dosing [4, 10] (Figure 2; Table 1).

**Table 1:** Technical comparison of the three cardioplegia strategies used for ALCAPA repair.

Parameter	Dual-root Custodiol^®^ (Technique 1)	Cold blood + Ostial (Technique 2)	Warm blood classical (Technique 3)
Base solution/ratio	Custodiol^®^ (HTK crystalloid) [1, 2]	St. Thomas II, 4:1 blood:crystalloid [3, 4]	Whole blood with customized K^+^/Mg^2+^ [5, 6]
Temperature at delivery	4–8 °C [1]	8–10 °C [3]	32–34 °C (systemic 28 °C) [5]
Primary delivery route(s)	Aortic root + pulmonary root (simultaneous) [1, 2]	Aortic root (initial) → direct LCA ostium via pulmonary stump [3]	Aortic root (repeating) → LCA ostium after MPA opening [5]
Initial perfusion pressure	80–90 mmHg (pediatric); 100–110 mmHg (adult) [1, 2]	100 mmHg throughout [3]	100 mmHg throughout [5]
Maintenance pressure post-arrest	40–50 mmHg [1]	80 mmHg root; 60 mmHg ostial [3]	100 mmHg continuous [5]
Dose (first shot)	20–30 mL/kg (max ~2 L) [1]	10 mL/kg root +5 mL/kg ostial [3]	8 mL/kg every 20 min [5]
Scheduled re-dose	None if clamp ≤ 90 min; 10 mL/kg at 90–120 min [1]	50% of initial dose every 30 min [3]	Full dose every 20 min; half-dose ostial every 40 min [5]
Ostial cannulation	Not required (simultaneous dual-root perfusion) [1]	Required: 3 Fr olive-tip cannula with 5–0 Prolene snare [3]	Required; same cannula with warm solution [5]
Protection window	120–150 min [1, 2]	25–30 min per dose [3]	15–20 min per dose [5, 6]
Typical LV distension grade	0–1 (if pressure controlled) [2]	0–1 [3]	1–2 (requires venting) [5]
Set-up time (estimated)	4–5 min (dual lines prepared pre-clamp) [1]	6–7 min (ostial cannula placed after MPA transection) [3]	3–4 min (single root line) [5]
Advantages	Single-shot, homogeneous protection; minimal redosing; effective for dual coronary bed [1, 2]	Precise LCA targeting; excellent for prolonged cross-clamp cases; blood buffer benefits [3, 4]	Familiar; real-time metabolic control; terminal warm reperfusion possible [5, 6]
Limitations	Requires pressure modulation; low viscosity risks LV overdistension; no metabolic flexibility [2]	Technically demanding ostial cannulation; dual circuit coordination needed [3]	Frequent dosing; increased OR workload; early rhythm recovery due to RCA-LCA collaterals [5, 6]

## Discussion

Effective myocardial protection during ALCAPA repair requires overcoming two unique challenges: the anomalous origin of the left coronary artery from the pulmonary trunk, which dilutes antegrade cardioplegia, and extensive right-to-left collateral circulation, which can provoke premature electrical recovery [9, 10]. Based on a narrative synthesis of PubMed-indexed pediatric cardioplegia studies and technical reports from MMCTS and CTSNet [11, 12], the three described strategies, dual-root Custodiol^®^, cold blood + ostial perfusion, and normothermic blood cardioplegia, each offer different trade-offs in balancing arrest duration, clarity of perfusion, and procedural complexity. Custodiol ^®^ delivers long-lasting myocardial preservation with a single dose, providing up to 2 h of protection by buffering intracellular pH and minimizing calcium influx. It is particularly effective when dual-root delivery is feasible, ensuring homogeneous perfusion with selective ostial cannulation [13, 14]. However, its low viscosity requires high initial perfusion pressures, later reduced to avoid ventricular distension. It is important to note that crystalloid cardioplegia in ALCAPA repair should not be interpreted as synonymous with Custodiol^®^ alone. Other crystalloid-based cardioplegia formulations, including modified del Nido and institution-specific solutions, have been reported in congenital cardiac surgery and may provide adequate myocardial protection depending on delivery strategy and dosing. However, crystalloid solutions may impose a greater systemic homeostatic burden, particularly in smaller pediatric and neonatal patients through hemodilution and electrolyte disturbances such as hyponatremia, which should be anticipated and actively managed during cardiopulmonary bypass. Most congenital centers currently favor blood-based cold cardioplegia, which offers superior oxygen-carrying and buffering capacity compared to crystalloid. Cold blood cardioplegia reduces reperfusion injury markers such as troponin release and improves metabolic recovery in pediatric patients, particularly those who are hypoxic or cyanotic. In the context of ALCAPA, the addition of direct ostial dosing after pulmonary artery transection ensures distal LCA bed perfusion that is not achieved by a single root dose [12]. While effective for prolonged cross-clamp periods, this strategy introduces technical demands in cannula positioning and coordinating dual perfusion timings. Warm blood cardioplegia presents the greatest intraoperative metabolic flexibility, allowing dynamic titration of electrolytes and use of a terminal “hot shot” to reduce reperfusion injury. The potential benefit of terminal warm reperfusion (“hot-shot”) in mitigating ischemia–reperfusion injury is largely extrapolated from broader congenital and adult cardiac surgery literature. At present, there is limited ALCAPA-specific evidence demonstrating a distinct protective advantage of hot-shot administration in this pathology, and its use should therefore be considered adjunctive and center-specific rather than pathology-driven [13]. In the context of abnormal coronary anatomy, such as ALCAPA, normothermic cardioplegia protocols warrant particular caution. Extensive coronary collateralization may predispose to early electrical activity and incomplete myocardial arrest, necessitating frequent redosing and vigilant rhythm monitoring. Accordingly, warm blood cardioplegia strategies should be employed selectively and preferably in centers with established experience in normothermic myocardial protection [12]. Pediatric randomized data, however, show equivalent biochemical and clinical outcomes compared to cold blood protocols, while adult series advocate its safety and ease once teams are experienced [14, 15]. The drawbacks are an increased workload due to frequent dosing intervals (15–20 min) and a persistent risk of early electrical return due to collateral flow.

### Practical considerations

- Single-dose crystalloid strategies may be considered when dual-root delivery is feasible and anticipated cross-clamp duration is moderate (operationally ≤90 min)**,** with careful control of perfusion pressure to minimize left ventricular distension.
- Cold blood cardioplegia supplemented by selective left coronary ostial perfusion may be advantageous during prolonged or staged repairs, allowing scheduled redosing and targeted myocardial distribution.
- Normothermic blood cardioplegia offers *metabolic control*, defined as real-time modulation of potassium concentration, temperature, hematocrit and oxygen delivery, and buffering capacity during repeated dosing; however, its use in ALCAPA requires careful rhythm surveillance due to the risk of early electrical recovery.
- In cases of incomplete electromechanical arrest, adjunctive myocardial protection strategies such as systemic hyperkalemia and deep hypothermia may be employed as rescue measures.

Since no randomized trials target ALCAPA specifically, decisions must rely on surgeons’ experience, anticipated cross-clamp times, and available perfusion infrastructure. Future multicenter studies should aim to compare these techniques head-to-head, focusing on myocardial injury markers, ventricular function, and long-term outcomes.

### Limitations

This review is subject to several limitations. First, the available literature consists predominantly of observational studies, technical reports, and case-based descriptions, with no randomized or comparative trials specific to ALCAPA cardioplegia strategies. As a result, no conclusions regarding the superiority of any myocardial protection technique can be drawn. Second, the review was limited to PubMed/MEDLINE, and relevant studies indexed in other databases may have been missed. Additionally, cardioplegia protocols are not uniformly reported, and institutional practices are often incompletely described, which may influence study selection and perceived prevalence of specific strategies. Finally, this review focused on technical aspects of myocardial protection and did not systematically analyze postoperative outcomes such as myocardial stunning, inotropic requirements, or postcardiotomy ECMO use, due to heterogeneity and inconsistent reporting across studies.

## Conclusions

ALCAPA repair presents unique intraoperative challenges in myocardial protection due to aberrant coronary anatomy and extensive collateral circulation. Through this narrative review, we identified and comparatively analyzed three cardioplegia techniques currently in clinical use, each demonstrating context-specific advantages. Simultaneous aortic and pulmonary root delivery of Custodiol^®^ crystalloid solution offers a streamlined, single-dose approach with extended protection and minimal need for redosing, proving ideal for procedures of moderate cross-clamp duration. Cold blood cardioplegia with ostial supplementation provides targeted perfusion to the left coronary territory and is especially suited for prolonged surgeries where staged myocardial protection is necessary. The normothermic blood strategy, while requiring more frequent redosing, affords real-time metabolic control and remains a reliable option in centers familiar with warm protocols. Ultimately, the optimal technique must be tailored to individual patient anatomy, institutional expertise, and the complexity of the repair. Surgeons should consider factors such as cross-clamp time, collateral flow patterns, and perfusion logistics when selecting a myocardial protection strategy, while acknowledging that the current evidence base is largely derived from observational reports, technical descriptions, and low-level non-comparative data. Further studies are warranted to better define optimal dosing intervals, solution volumes, and pressure targets specific to pediatric ALCAPA repair. Establishing standardized protocols supported by prospective data could significantly enhance myocardial protection and long-term outcomes in this fragile patient population.

## Data Availability

All data supporting the findings of this study are available from the corresponding author upon reasonable request.
